# Verbal Fluency Deficits Co-Occur with Memory Deficits in Geriatric Patients at Risk for Dementia: Implications for the Concept of Mild Cognitive Impairment

**DOI:** 10.3233/ben-2009-0246

**Published:** 2010-06-29

**Authors:** Maria E. Cottingham, Keith A. Hawkins

**Affiliations:** ^1^Yale University School of MedicineNew HavenCTUSA; ^2^Harbor-UCLA Medical CenterTorranceCAUSA

**Keywords:** Mild cognitive impairment, MCI (amnestic), Alzheimer's Disease, verbal fluency, phonemic fluency, semantic fluency, memory, neuropsychological, cognition, dementia

## Abstract

We tested the notion that patients at high risk for progression to Alzheimer's disease (AD) display relatively isolated memory deficits by assessing the relationship between memory and fluency performances in a sample of 92 geriatric subjects with cognitive complaints and normal to mild clinical presentations. Patient groups were formed on the basis of memory test scores. Patients with normal memory scores also performed normally on fluency tests, and their fluency scores were significantly higher than those of patients with low memory performances. Patients falling between these two groups in memory abilities also displayed intermediate level fluency performances. Whereas the normal memory group performed at equivalent levels on semantic and phonemic fluency tasks, both the impaired memory group and the intermediate group displayed relatively greater weaknesses in semantic fluency. This pattern is similar to that seen in AD. Since the impaired memory patients meet criteria for Amnestic Mild Cognitive Impairment, these findings suggest that memory deficits in “pre-clinical” AD are likely to be accompanied by fluency weaknesses, with semantic fluency weaknesses predominating.

### 1. Introduction

The term Mild Cognitive Impairment (MCI) has found an ever-widening currency within the literature to denote a heightened risk for dementia in patients who are not demented at the time of their examination [41]. As initially construed by Petersen and colleagues [36,38,40], MCI was intended to identify preclinical Alzheimer's disease. Non-demented patients meet criteria if they remain independent in activities of daily living, yet have cognitive complaints and display impairment on formal memory tests [38,41]. Patients diagnosed with MCI by these criteria have progressed to AD at annual rates between 6 and 25% [41].

Within a growing literature on states that fall between normal functioning and dementia, a debate has emerged regarding the true nature of MCI, progression rates, and eventual clinical status ([4,6,11,12,15, 16,18,23,27,30,32,42,43,46,49]). Petersen and his colleagues have accordingly refined their conception of MCI over the years to include four subtypes: Amnestic MCI Single Domain; Amnestic MCI Multiple Domain; Non-Amnestic MCI Single Domain; and Non-Amnestic MCI-Multiple Domain [28,39]. According to Petersen, progression within [8] either Amnestic subtype will likely result in a diagnosis of AD, but vascular dementia or depression may also cause the MCI presentation. Non-Amnestic MCI may reflect fronto-

ISSN 0953-4180/09,10/$27.50 © 2009/2010 – IOS Press and the authors. All rights reserved temporal, Lewy body, or vascular etiologies, though these causes are considered largely theoretical [28].

Though the conception of MCI had broadened, Petersen and colleagues continue to stress the pre-eminence of memory deficiencies in the presentations mostly likely to progress to AD [28]. Since they expect memory deficiency to be the cardinal sign of an AD risk state, they have recommended that practitioners supplement their clinical and brief cognitive status exams with a memory test (such as word list or paragraph recall) [31]. Other research, though, indicates that broader deficiencies are likely to be present in "pre-clinical" AD [4], raising the possibility that other brief measures warrant consideration for screening purposes. There is evidence that the presence of additional cognitive deficiencies raises the probability of conversion to AD, i.e. improves the diagnostic accuracy of MCI (insofar as the diagnosis designates a risk state) [8].

Verbal fluency weaknesses have frequently been reported [2,3,7,10,14,18–20,22,23,26,31,32,35,42,44, 45,48]. Since fluency measures are brief and easily administered, they are ideal for "bedside" or office-visit screening purposes. If fluency deficits are in fact common in pre-clinical AD, the consideration of fluency scores in addition to memory scores could possibly enhance the identification of true positives for progression [1,8].

Though there have been negative fluency findings in MCI research, these findings have not been compelling. Goldman et al. [17] found no weakness in fluency in a group with confirmed pre-clinical AD when compared with healthy controls, but restricted their fluency measurement to production to two letters, and their MCI group was very small ($n = 5$). Lambon Ralph et al. [29] found patients with MCI performed similarly to a control group on tasks of phonemic fluency; they did not, however, assess semantic fluency, which is more commonly depressed in AD. Another study [27] found that though verbal fluency performances did not statistically differ between MCI and control subjects, or between MCI and AD patients, the differences were in the expected direction and study design had limited statistical power (MCI $n = 15$). The question of fluency deficits as a prognostic marker (for progression to AD), or as a diagnostic feature of MCI, therefore remains an open one.

A related question pertains to the possibility of a differential in fluency performances between phonemic (production to letter) and semantic (production to category) fluency. In established AD, semantic fluency test scores are generally lower than phonemic fluency scores [9,21,33,34]. To the best of our knowledge, the possibility that a similar discrepancy exists in MCI has not been investigated.

The possession of archival geriatric neuropsychological data afforded us the opportunity to determine whether memory impairments are accompanied by fluency weaknesses in geriatric patients with cognitive complaints. The patients forming this dataset were referred for a neuropsychological assessment because of diagnostic uncertainty, typically due to the presence of questionable or mild clinical features. Approximately 90% of patients seen in the clinical setting within which these patients were assessed do *not* undergo formal testing (most satisfy diagnostic criteria for dementia, or related states, on clinical grounds alone). This attests to the mild to normal clinical status of the patients making up this neuropsychological sample.

Given the presence of memory complaints among these patients, and the fact that AD is the most common cause of dementia, it is reasonable to presume that many of these patients are true MCI-Amnestic cases (i.e., that a significant portion will progress to AD). We opted to test the notion that isolated memory deficits occur by examining the relationship between memory and fluency performances within this sample. Along with allowing us to determine whether deficits in fluency co-occur with memory deficits, these data also allowed for the exploration of the relationship between semantic and phonemic fluency. Specifically, we addressed the following hypotheses:

1. Increasing levels of memory impairment will be accompanied by increasingly deficient fluency performances.
2. Patients with normal memory performance will exhibit a normal relationship between the fluency measures.
3. Patients with evidence of memory impairment will show greater relative levels of impairment in semantic fluency than in phonemic fluency.

### 2. Methods

#### *2.1. Participants*

The participants were 92 patients with cognitive complaints referred for neuropsychological assessment by geriatricians. Exclusion criteria included: a non-AD dementia diagnosis; a diagnosis of bipolar disorder or schizophrenia; primary language other than English; lack of independence in activities of daily living; age

Table 1
Demographic and performance variables by group

| Memory: psychometric status | Age | Education | WRAT reading Std score | MMSE | WMS-III word list total recall (ss)[1] | WMS-III delayed recall (ss)[1] | WMS-III word list recognition (raw) | Phonemic fluency: FAS Raw Score | FAS: mean per letter | Semantic fluency: Animals | Phonemic fluency: Z score (Normed) | Semantic fluency: Z score (Normed) |
|---|---|---|---|---|---|---|---|---|---|---|---|---|
| Normal | 67.8 | 16.5 | 113.6 | 29.4 | 12.6 | 13.3 | 23.2 | 47.1 | 15.7 | 21.1 | 0.5 | 0.6 |
| ($n = 17$) | (8.6) | (2.7) | (6.0) | (0.9) | (1.9) | (2.0) | (0.8) | (9.6) | (3.2) | (4.0) | (0.8) | (0.7) |
| Intermediate | 75.0 | 14.7 | 108.2[2] | 27.8 | 7.0 | 9.4 | 18.7 | 35.5 | 11.8 | 14.2 | −0.3 | −0.7 |
| ($n = 54$) | (6.8) | (3.1) | (9.4) | (1.8) | (2.4) | (2.3) | (3.0) | (11.8) | (4.0) | (5.2) | (1.0) | (1.1) |
| Impaired | 70.3 | 14.4 | 104.6 | 26.3 | 3.9 | 6.0 | 15.5 | 32.1 | 10.7 | 12.7 | −0.6 | −1.2 |
| ($n = 21$) | (5.7) | (2.5) | (8.1) | (2.4) | (1.8) | (0.0) | (2.1) | (11.0) | (3.7) | (4.8) | (1.0) | (1.2) |

*Note.* Numbers in parentheses are standard deviations.
[1]ss = Age-Corrected Scaled Score; [2]$n = 51$.

under 50 years. The sample was 96% Caucasian and 51% male. The mean age was 72.6 years (SD = 7.5, range = 51–90). Mean education was 14.9 years (SD = 3.0, range = 8–20).

Participants were separated into three groups based upon performances on the Wechsler Memory Scale-III [13]: normal, borderline to mild impairment, and memory impaired. The normal group scored at normatively average levels or higher on the WMS-III Word List total recall score (sum of recall for trials 1 through 4) and on the delayed recall portion of the test (i.e., on both immediate and delayed recall they attained a scaled score of 10 or higher, where 10 is equal to the average score for similarly aged normal subjects). In addition, to be considered to exhibit normal memory, the participants also had to score 22 or higher out of 24 on the Word List recognition trial.

In contrast, participants were considered to display psychometric memory impairment if their scaled scores for the Word List for both immediate and delayed recall were 6 or lower, and their recognition scores were 18 or lower. Because a scaled score of 6 typically denotes a performance that falls about 1.33 standard deviations below the mean, and since we required scores of 6 or less on both immediate and delayed recall along with a low recognition score, the overall list memory performances of this group falls well below 1.5 standard deviations below the normal population mean.

All participants not meeting criteria for either the psychometrically normal or impaired groups were assigned to an intermediate group. Though the mean Word List scaled score for this group falls at 1 standard deviation below average ($M$ = 7.04, $SD$ = 2.39), they show a significant discrepancy between their mean WRAT Reading scores and their memory scores when both are transformed to z scores (see below; $M = -1.53$, $SD = 0.92$), $t$ (50) $= -11.98$, $p < 0.001$. Given the correlation between reading and memory scores in normal populations [37], it is reasonable to presume that the memory functioning of this intermediate group has declined significantly from premorbid levels. Demographic and performance data for all three groups are presented in Table 1.

#### 2.2. Measures

##### 2.2.1. WMS-III word lists [44]

The WMS-III Word List test requires learning a list of words over 4 trials, recalling the list after a distraction list, and then recalling the list again after a 30-minute delay. Participants are then asked to identify the words from a list consisting of the words plus an equal number of foils (recognition trial). Scaled scores for total list recall over the four learning trials, and for delayed recall, were acquired via normative data provided in the WMS-III manual. Raw scores were used for the recognition component of the test, as scores for this trial are not normally distributed. The normative data for this test are age stratified. For graphing purposes, so that data are directly comparable with fluency and reading scores, Word List I total scaled scores were transformed into z scores based on the relationship between WMS-III scaled scores and the normal curve.

##### 2.2.2. Verbal fluency

To assess phonemic verbal fluency, participants were asked to name as many words as possible within 60 seconds that began with various letters of the alphabet across 3 trials [5]. They were also asked to name as many animals as possible within 60 seconds in order to assess semantic verbal fluency. To examine the relationship between phonemic and semantic fluency performance relative to a normal sample, both fluency scores were converted to standard z scores [(raw score-$M$)/$SD$] using normative data stratified by age and education [47]. Figure 1 shows the normed variable means by group.

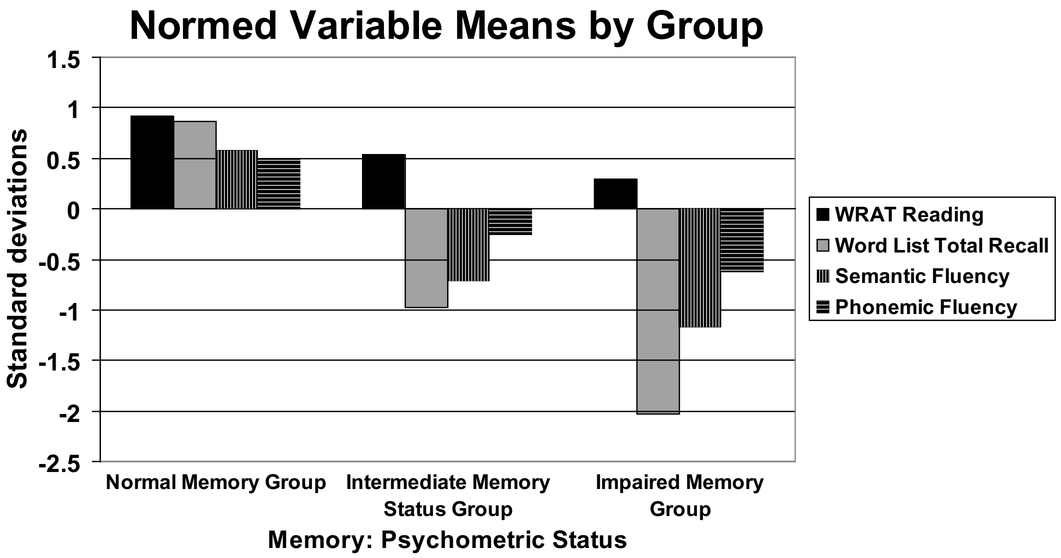

Fig. 1.

*2.2.3. Wide range achievement test, reading (WRAT)*

Participants' reading scores, a strong correlate of premorbid status and educational attainment, are presented to characterize the sample. Participants had either WRAT-R [24] or WRAT-3 [25] data; the tests are highly similar. So that data would be directly comparable to fluency and memory scores, WRAT standard scores were transformed into standard z-scores [(Reading SS-100)/15].

### 2.3. Data analysis

All analyses were conducted by using computerized statistical software (SPSS 15.0, Chicago, IL). For all statistical tests, the level of significance was one-tailed, 0.05.

*2.3.1. Hypothesis 1*

Between-group differences among raw fluency performances were examined by use of analysis of variance (ANOVA). A post-hoc Tukey's Honest Significant Difference test inspected the specific differences. An analysis of covariance (ANCOVA) with a demographic covariate (age) and general intellectual ability covariates (MMSE score, education) was also run. In addition, correlations between raw fluency scores and raw Word List I total scores were run, partialing out age, education, and MMSE. Finally, to control for group differences in premorbid general cognitive ability, an analysis of covariance (ANCOVA) with reading score as the covariate was run for each type of fluency.

*2.3.2. Hypothesis 2*

A paired t-test was run to compare the mean difference between normative scores for semantic and phonemic fluency in the normal memory group.

*2.3.3. Hypothesis 3*

Paired t-tests were performed to compare the mean difference between the two fluency variables, expressed as normative scores, for the intermediate and impaired memory groups.

## 3. Results

### 3.1. Hypothesis 1

Between-group differences were found for phonemic fluency, $F(2, 91) = 9.16$, $p < 0.001$. Post-hoc testing showed significant differences between the normal and intermediate groups ($MD = 3.85$, $SD = 1.05$, $p < 0.001$), and between the normal and impaired groups ($MD = 4.97$, $SD = 1.23$, $p < 0.001$). In addition, between-group differences were found for semantic fluency, $F(2, 91) = 16.00$, $p < 0.001$. Post-hoc testing revealed significant differences between the normal and intermediate groups ($MD = 6.91$, $SD = 1.37$, $p < 0.001$) and between the normal and impaired groups ($MD = 8.34$, $SD = 1.60$, $p < 0.001$). After controlling for age and education, the overall result remained significant for both phonemic, $F$ (4, 91) $= 8.03$, $p < 0.001$, and semantic fluency, $F$ (4, 91) $= 15.44$, $p <$

0.001. Furthermore, raw scores for both phonemic, $r$ = 0.49, $p <$ 0.001, and semantic, $r$ = 0.45, $p <$ 0.001, fluency remained significantly correlated with Word List I total raw scores after partialing out age and education.

An analysis of covariance was undertaken to determine whether the groups differed in fluency after controlling for differences in reading score, a proxy for general ability. After controlling for reading score, the differences in phonemic fluency between the groups remained, $F$ (2,85) = 9.07, $p <$ 0.001. Controlling for reading score with semantic fluency as the dependent variable produced a similar result, $F$ (2,85) = 15.46, $p <$ 0.001.

We also ran an analysis of covariance to assess whether the normal group exhibited higher memory scores after group differences in reading score were controlled for. After controlling for reading score, the differences in memory between the groups remained, $F$ (2,85) = 65.5, $p <$ 0.001. Neither the lower memory scores nor the lower fluency scores of the lower performing groups are explainable on the basis of their lower reading scores; the differences in memory and fluency between the groups likely reflects declines in these functions rather than pre-existing differences in ability.

Neither phonemic nor semantic fluency correlated with memory significantly in the normal memory group ($r$ = 0.21, ns, $r$ = 0.27, ns for FAS and Animals, respectively). In patients showing mild memory impairment or worse, memory correlated with fluency, $r$ = 0.47 (FAS), $p <$ 0.001 (FAS) and $r$ = 0.35, $p <$ 0.01 (Animals). This suggests that memory and fluency both decline with the onset of dementia, with each potentially indexing overall status.

Figure 1 shows that, whereas the reading scores for the groups are comparable, the fluency scores for the memory impaired groups are substantially lower.

#### 3.2. *Hypothesis 2*

As hypothesized, normative scores for semantic and phonemic fluency did not differ in the normal group ($M = -$0.06, $SD$ = 1.02), $t$ (16) = $-$0.26, $p$ = 0.80.

#### 3.3. *Hypothesis 3*

The mean difference between the two fluency variables was significant for both the intermediate ($M$ = 0.46, $SD$ = 1.36), $t$ (53) = 2.50, $p <$ 0.01, and the impaired ($M$ = 0.55, $SD$ = 1.09), $t$ (20) = 2.31, $p <$ 0.05, groups.

### 4. Conclusions

We found that increasing levels of memory impairment were accompanied by increasingly deficient fluency performances in patients with normal to mild clinical presentations who were referred within a geriatric clinic for neuropsychological assessment. In addition, whereas patients with normal memory performance exhibited a normal relationship between semantic and phonemic fluency, patients with moderate to significant memory impairment displayed greater levels of impairment in semantic fluency than in phonemic fluency (Fig. 1). A recent comprehensive meta-analysis revealed that this pattern predominates in AD [21]; our finding is therefore unsurprising, but also novel insofar as the question has been rarely addressed in MCI and other nuanced presentations.

The patients comprising the sample had cognitive complaints, but were considered not to be diagnostically clear-cut due to their mild, and sometimes normal, clinical presentations. When patients in this and similar geriatric settings exhibit a degenerative disorder, the most common eventual diagnosis is AD. In the setting of this study, patients with symptoms of a movement disorder, or of stroke, are likely to be referred to other clinics, and/or are unlikely to be referred for a neuropsychological work-up within the geriatric clinic. Patients exhibiting significant cognitive deficiencies are also unlikely to be referred for a neuropsychological exam. In short, we believe that the patients within our sample typically would not meet criteria for dementia, but those with memory deficits do meet criteria for Amnestic MCI, and many of these will develop AD. Patients with memory deficits in our sample also performed poorly on fluency measures, suggesting that memory deficits may only infrequently occur in isolation in "pre-clinical" AD cases. Our data also suggest that in patients at heightened risk for AD, semantic fluency is more commonly depressed than phonemic fluency.

MCI has proven to be more complex than initially thought [37,39], and our finding supports ongoing refinement to the concept. It is possible that single domain and multiple domain amnestic MCI reflect a qualitative continuum rather than truly distinct subtypes. Petersen, an early proponent of diagnostic criteria that specified an isolated memory deficit, has modified his formulation to include both single and multiple domain versions of Amnestic MCI [28]. This in itself represents a modification of a trend within the literature towards recognition of two major subtypes,

MCI-Amnestic and MCI-Multiple Domain, insofar as it recognizes that memory weaknesses may be accompanied by deficits in other domains in patients who are, nonetheless, at risk for progression to AD (rather than some other form of dementia). Our data support the notion that Amnestic MCI-Multiple Domain presentations are commonplace within the universe of patients at heightened risk for AD.

Though in part a semantic issue, “pure” MCI amnestic cases may be rather rare. The initial model of MCI, with a single domain (memory) deficit in the context of normal broader cognition, possesses heuristic value, but may have been a simplification that has survived in part because “normal” has been interpreted loosely. If patients are defined on the basis of a relatively prominent memory deficit, performances have to be primarily higher in other domains. Readers will note that within the memory impaired group the mean memory deficit exceeds the mean semantic fluency deficit (Fig. 1). This is an artifact of the grouping method, i.e., because the subjects were grouped on the basis of memory performance, the memory deficit of the impaired group will, *ipso facto*, exceed any other deficiency. The fluency scores in this group are notably weaker than their reading scores, a measure more resistant to decline with brain compromise (and a strong fluency correlate in healthy subjects). These data make it clear that these memory-compromised patients have also suffered losses in verbal fluency. Weaknesses in non-memory domains in similar samples, or in individual cases, will be over-looked if all performances falling above the threshold set for the memory data (whether one or 1.5 standard deviations below the mean) are considered “normal”. The presence of deficits in multiple domains has been predictive of higher AD conversion rates in several studies [8,43], further underscoring the possibility that early AD cases suffer declines in more than just memory.

A limitation of our study is that it was cross-sectional in nature, and we have no knowledge of final diagnostic outcomes. However, given the nature of the referral setting, and the high base rates for AD within dementia, it is likely that sizeable portions of our intermediate and impaired memory groups will develop AD. Further research will be required to determine whether consideration of fluency performances in addition to memory deficits facilitates the identification of “true positive” early AD cases among patients with memory complaints.

### Acknowledgements

This work was supported in part by an Alzheimer’s Association Investigator Initiated Award (IIRG-05-14532) to Keith Hawkins.
